# Bacterial degrons in synthetic circuits

**DOI:** 10.1098/rsob.220180

**Published:** 2022-08-17

**Authors:** Prajakta Jadhav, Yanyan Chen, Nicholas Butzin, Javier Buceta, Arantxa Urchueguía

**Affiliations:** ^1^ Department of Biology and Microbiology, South Dakota State University, Brookings, SD, USA; ^2^ Program for Computational and Systems Biology, Memorial Sloan Kettering Cancer Center, New York, NY, USA; ^3^ Institute for Integrative Systems Biology (I2SysBio, CSIC-UV), Paterna, Valencia 46980, Spain

**Keywords:** degradation, queueing theory, proteases, oscillatory circuits

## Abstract

Bacterial proteases are a promising post-translational regulation strategy in synthetic circuits because they recognize specific amino acid degradation tags (degrons) that can be fine-tuned to modulate the degradation levels of tagged proteins. For this reason, recent efforts have been made in the search for new degrons. Here we review the up-to-date applications of degradation tags for circuit engineering in bacteria. In particular, we pay special attention to the effects of degradation bottlenecks in synthetic oscillators and introduce mathematical approaches to study queueing that enable the quantitative modelling of proteolytic queues.

## Graphical abstract

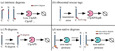

## 1. Introduction

The increased ability to engineer genetic networks [1] has enabled the construction of various synthetic circuits, such as the toggle switch [2], Boolean-like gates [3,4] and circuits exploiting positive/negative feedback loops [5,6]. These circuit topologies mimic the core behaviour of natural gene networks and provide organisms with new functionalities [7–10]. Notably, most of these circuits rely on transcriptional regulation. Taking the cue from natural systems, which often use transcription, translational and post-translational regulation to fine-tune outputs (often protein levels), researchers have recently leveraged all three in new circuits [11–13]. In particular, proteases have been leveraged to regulate the behaviour of synthetic circuits at the protein level to increase circuit control and output in bacteria [13,14] and eukaryotes [15–17]. Cellular proteases recognize specific amino acid sequences, known as degradation tags or degrons [14], and are crucial in maintaining the homeostasis of proteins [18,19]. Degrons can be of various lengths intrinsically present in the protein’s sequences, result from the ribosomal rescue system tagging the C-terminus [20,21], or caused by the enzymatic modification of the N-terminus of a protein [22–25]. Proteins tagged with degrons have faster and tunable degradation rates compared to untagged proteins due to the degradation activity of proteases. As far back as the 1960s, seminal theoretical models highlighted the importance of controlling the degradation rates to obtain robust circuit outputs, particularly in synthetic oscillator designs [26,27].

Synthetic circuits often use heterologous (foreign) proteins or highly produced host proteins. Target proteins are commonly induced by tightly regulated promoters using high copy number plasmids to have robust control and maximize output. Metabolically speaking, this can result in cellular burden [28–31], which is only exacerbated because heterologous proteins accumulate cellular space (e.g. cells have a finite cytoplasm) and their amino acids are not recycled (loss of energy). Without the use of degradation tags protein turnover is dependent on dilution from growth/division. This process can be slow and, importantly, highly dependent on the strain and environment. For example, slower-growing cells will have more buildup of heterologous proteins than faster-growing cells. Moreover, the lack of controlled degradation may lead to coupling effects between the circuit’s output, its regulation and the host growth rate [32–35]. Ultimately, these effects limit the complexity, temporal resolution and scalability of the design of synthetic circuits.

In this context, bacterial degrons are a key part of the synthetic biology toolbox [36,37]. Degradation tags add an extra layer of circuit regulation and provide a handy post-translational method to uncouple the growth rate from a circuit’s output. In addition, degradation tags decrease cellular burden because heterologous proteins accumulate less in the cytoplasm (taking up less cellular space), and their amino acids can be recycled (less loss of energy). Yet, their importance and potential are still being unravelled, largely because the available ‘palette’ of degradation tags and proteases in synthetic biology is still limited. To date, most bacterial synthetic circuits exploit almost exclusively one *E. coli* degron: the SsrA tag (*ec*-SsrA) and its variants [37,38]. This tag is primarily recognized by the ClpXP proteolytic complex [39].

A limitation of using one protease system is that proteases are naturally maintained at limited numbers to achieve a tight regulation of the proteome machinery. When high levels of proteins are produced, degradation bottlenecks can develop even if proteins are tagged and lead to the formation of proteolytic queues [40,41]. The importance of proteolytic queueing to the output of many synthetic circuits has often been disregarded/underappreciated. Proteolytic queueing has mostly been studied in synthetic oscillators [42], a circuit topology where degradation tags are particularly relevant since protein expression is regulated by production/degradation waves. For example, queueing has been leveraged to couple otherwise unrelated circuits [11,43,44]. Coping with limited resources also leads to redundancy in degradation pathways. That is, when ClpXP becomes saturated, a fraction of its targets can be degraded by other proteolytic complexes [41,43]. On the other hand, it can also be a nuisance when designing a robust degradation strategy in synthetic circuits, as it hinders the ability to precisely control degradation rates. From a theoretical perspective, although current models take into account enzymatic degradation [45,46], they do not typically incorporate the effects of degradation bottlenecks to understand circuit’s dynamics except in very few studies [11,41,47].

New circuits with degradation tags targeted to other proteases beyond ClpXP have been developed in the last few years [43,48–50]. We also now have a better understanding of how finite processing resources (e.g. proteases) regulate the behaviour of complex circuits [14,48,51]. In particular, the connection between processing pathways in a biological context and theoretical frameworks—classically applied to computer systems and call centres—has paved the way to understand how queueing affects the output of synthetic biological circuits [52].

In this review, we focus on the use of bacterial degradation tags (degrons) in synthetic biology for applications in prokaryotic systems. First, we discuss currently used degradation tags and recent advances in the search for alternative degrons and their potential benefit to construct new circuits. Specifically circuits with minimal crosstalk between proteolytic pathways. Second, given that queueing effects have been more clearly revealed/understood in oscillatory circuits, we review recent research showing how proteolytic bottlenecks affects the output of synthetic oscillators. Finally, we introduce the mathematics of queueing theory and the approaches typically used to model systems with bottlenecks. Altogether, by emphasizing the role played by degrons in ‘oscillatory synthetic biology’ and by introducing the theoretical and modelling frameworks of queueing, our review supplements recent efforts in the field [14] that highlight the uses of bacterial degrons in the context of synthetic applications and as novel antimicrobials.

## 2. Bacterial degrons: from natural systems to circuit engineering

### 2.1. Main degradation pathways in bacteria

**Figure 1.**
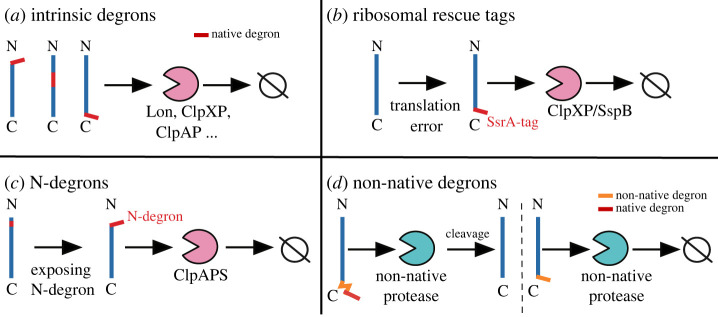
Examples of native (*a*–*c*) and non-native (*d*) degrons. (*a*) Intrinsic degrons can be present anywhere along the protein sequences. Different proteases can recognize these sequences [43]. (*b*) Ribosomal rescue tags (i.e. SsrA tag) are added at the C-terminal of proteins because of translational errors. The SsrA tag is mainly degraded by the proteolytic complex ClpXP [54]. The degradation of SsrA tagged proteins can be further enhanced by the chaperone SspB, which carries proteins to ClpXP [55]. (*c*) ClpAPS is the main proteolytic complex that recognizes N-degron tags at the N-terminal of proteins. These specific degrons can be hidden and only exposed by specific enzymes such as chaperones [20]. (*d*) A common strategy in synthetic biology is to fuse non-native degrons to proteins to reduce their half-life [56]. Left: A protein is unstable because it contains a degron that targets it to be degraded. However, the expression of the non-native protease can lead to protein stabilization because this protease cleaves off the degron sequence. Right: The target protein is stable until the induction of a non-native protease, which then degrades the target protein after recognizing a specific non-native degron.

In bacteria, three major degradation signalling systems exist: intrinsically present in a protein’s sequence [25] (figure 1*a*), the ribosomal rescue system tagging the C-terminus of a peptide [20,21,23] (figure 1*b*) and enzymatic modifications of the N-terminus of a peptide [20,22,53] (figure 1*c*).

Proteases are able to recognize these degradation signals and bind to the tagged proteins, which leads to their unfolding and degradation [57]. Proteases typically display different binding affinities for different tagging systems [43], providing a post-translational regulation mechanism to modulate protein levels. The degradation signal can be located at any position along protein sequences, and we call these intrinsic degrons (figure 1*a*). A properly folded protein is protected from proteases since the surrounding amino acids *hide* the degrons [25,58–63]. Intrinsic degrons are exposed by external signals, such as stress, or by the action of chaperones [64], and can be recognized by a specific protease or by multiple proteases.

The most extensively studied bacterial degradation tag is the SsrA-tag, located at the C-terminal of proteins [54]. It originates from the trans-translational rescue system (ribosomal rescue system [21,65]), which processes defective proteins due to translational errors [66–68] (figure 1*b*). This system frees stalled ribosomes to maintain the ribosomal pool and conserve cell functionality by adding the SsrA degradation tag to the C-terminal of unfinished polypeptides. These peptides are then degraded primarily by ClpXP in most bacteria [21,38,69–77]. While bacterial species can show variations in their SsrA-tag sequences [78–81], the tag commonly ends with a C-terminal motif of three amino acids, LAA. However, Mollicutes have a conserved ending NYAFA motif on their C-terminal tags mainly recognized by the protease Lon [82].

**Figure 2.**
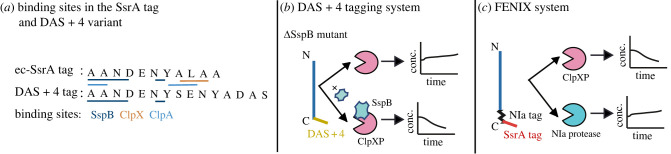
(*a*) Native *E. coli* SsrA tag and the DAS+4 variant. The underlying line indicates known binding sites for different *E. coli* chaperones and proteolytic units in the respective amino acid sequences (SspB: dark blue, ClpX: orange, ClpA: light blue) [81,83]. (*b*) Schematic representation of the DAS + 4 tagged-based system [83]. In the absence of SspB (e.g. in a ΔSspB mutant), DAS + 4 tagged proteins are stable, and after SspB is expressed the target proteins are degraded by ClpXP. (*c*) The FENIX system [84] is based on a SsrA/NIa hybrid tag. Active degradation is mediated after recognition of the SsrA tag by ClpXP. When the non-native NIa protease is not expressed, the target protein is stable. When NIa is produced, it cleaves at its recognition site resulting in the removal of the SsrA tag, leading to a stable protein level.

The most studied SsrA tag is from *E. coli* (*ec*-SsrA), and it consists of an 11 amino acid sequence (AANDENYALAA) that includes a binding site for the chaperones ClpX, ClpA and SspB [39,81] (figure 2*a*). ClpX and ClpA are unfoldases and members of the AAA+ family of proteins (ATPases) [85] that, after unfolding polypeptides in an ATP-dependent manner, transfer the tagged peptides to the caseinolytic protease ClpP. ClpP breaks the polypeptide bonds releasing free amino acid monomers [86,87]. ClpXP is the major proteolytic complex that recognizes *ec*-SsrA [39,88,89], and its activity can be further modulated by the chaperones SspB [55,90,91] and ClpS [92]. SspB binds to the *ec*-SsrA tag and then caries it to ClpX increasing ClpX’s specificity to the tag. By contrast, ClpS binding to ClpA reduces ClpA interactions with *ec*-SsrA tags [92], and enhances its role in the degradation of proteins with specific N-degrons [93,94] (figure 1*c*). Although the *ec*-SsrA tag is primarily recognized by ClpXP, it has a low affinity to other proteases such as ClpAP, Lon, FtsH and Prc (also named Tsp) [38,39,64,95–98]. When multiple proteases recognize the same tag, it can lead to proteolytic crosstalk. This crosstalk is particularly important in synthetic circuits using multiple degradation tags, as it hinders the ability to tightly control the protein levels and prevents orthogonal (i.e. uncoupled) circuits from being built [43].

We have already covered the basics of the C-terminal degradation tag, but the N-terminal residues (N-degrons) can also affect a protein’s half-life [20,99,100] (figure 1*c*). The N-degron pathway is universal, although in bacteria it is best understood in *E. coli* [20]. In *E. coli*, ClpAPS is the main proteolytic complex recognizing N-terminal residues for degradation [20,94]. The N-terminal destabilizing amino acids have been divided into primary degrons (1^o^-degrons: Leu, Phe, Tyr and Trp) and secondary degrons (2nd-degrons: Arg and Lys) [101]. ClpS can directly recognize primary degrons. By contrast, secondary degrons require the action of an amino acid transferase that facilitates ClpS binding by adding a primary degron sequence to the N-terminus [93,94,102,103]. For example, the L/F-transferase can modify the N-terminus of proteins by adding a Leu or Phe amino acid to a secondary degron [104]. N-degrons hold potential for expanding the synthetic biology toolbox, although their use is still limited [15,50,56,105].

### 2.2. Engineering degradation signals

The SsrA-tag was first discovered in *E. coli* (*ec*-SsrA) in 1995 [36] and was later used to study protein kinetics (degradation rates and protein turnover) [37]. The tag and variants (LVA, AAV and SVA), mutation of the last three critical residues (LAA), were fused to the C-terminal of fluorescent proteins [37,38]. Most synthetic circuits to date, especially dynamic circuits, exploit almost exclusively this tag and variants for targeted degradation. Apart from *E. coli*, synthetic circuits using the tag have been tested in other bacterial species, including bacillus [106,107], pseudomonas [108], salmonella [109], mycobacteria [110–112] or cyanobacteria [113]. The most common design includes fusing the SsrA tag to the C-terminal of the proteins of interest [37]. However, the lack of degradation specificity of the tag by ClpXP (i.e. binding sites that can be recognized by other proteases; figure 2*a*) still hinders the ability to control the degradation levels tightly in many circuits. New designs are required to make degradation systems that are more robust in their control and portable between bacteria.

One approach has been to leverage the specificity of the chaperone SspB to enhance the degradation by ClpXP using a modified degron tag known as DAS [83]. The DAS variant consists of a mutated tag in which the last three conserved amino acids (LAA) have been changed to DAS to minimize ClpX binding [83] (figure 2*a*). DAS has been further improved by linking four additional amino acids (SENY) to the rest of the native tag (Das+4 tag: AANDENY-SENY-ADAS; figure 2*a*). The degradation of the DAS + 4 variant by ClpXP in *E. coli* occurs effectively when SspB is induced [83] (figure 2*b*). This strategy has also been successfully implemented in *B. subtilis* [106] and mycobacteria [110]. The DAS + 4 tag-based system was further modified by implementing a split adaptor system [114], where SspB is split into two domains, SspB*CORE* and SspB*XB*. A functional SspB protein only forms in the presence of the antibiotic rapamycin, thus enabling an additional layer of control for the DAS + 4 tag degradation [115,116].

A different approach to improving the degradation specificity of the *ec*-SsrA tag is the use of hybrid tags with cleavage sites identified by viral proteases [56] (figure 1*d*). While the use of degrons recognized by proteases allow for the direct regulation of protein degradation levels, viral proteases typically recognize and cleave specific peptides from a protein sequence [117,118]. Thus, they are not directly involved in the degradation of proteins; when used in combination with degrons they can improve protein stability [15,50,56,119,120]. In bacteria such a strategy has been implemented in the *FENIX* system (Functional Engineering of SsrA/NIa-based fluX control) which uses a hybrid *ec*-SsrA/NIa (viral nuclear inclusion protein A) tag [84] isolated from the turnip mosaic potyvirus [121] (figure 2*c*). In the absence of a NIa protease, FENIX allows for active degradation of the *ec*-SsrA-tagged protein by ClpXP. However, expression of the NIa protease results in the NIa-tag being cleaved, causing the removal of the SsrA-tag from the target protein and stabilizing its levels [84]. FENIX has been successfully used to uncouple the production of biopolymers to growth rate [84], and decrease leaky gene expression in *P. putida* [108].

Another innovative approach is to use the natural variation of the SsrA tag sequence in different bacteria. The SsrA tag sequence is conserved in most bacterial species [81]; however, Mycoplasma species (class Mollicutes) have evolved a different SsrA amino acid sequence because they lack an active ClpXP proteolytic complex [82]. The SsrA tag from *Mesoplasma florum* (*mf*-SsrA) is longer than its *E. coli* analogous, and ends with a NYAFA motif recognized by the *M. florum*’s Lon protease (*mf*-Lon) [48,82]. The Collins’ group has explored the usage of *mf*-SsrA variants for synthetic biology applications by cloning proteins with this tag in *E. coli* [48]. Two variants have been tested in synthetic circuits. In these circuits, the degradation of tagged proteins is driven by *mf*-Lon co-expression [122,123]. However, some studies showed that *mf*-SsrA tagged proteins can escape most, but not all, endogenous *E. coli* proteases [82,124]. Some efforts have been made to improve *mf*-Lon’s specificity by systematically deleting particular residues [125] in one of the *mf*-SsrA variants created in [48]. Another concern is that non-native proteases may add metabolic burden on the host by targeting native proteins, thus affecting large protein networks. With that being said, the approach of using a non-native tag has significant potential to allowing the design of complex orthogonal circuits within the same host. The Sauer’s group explored this and showed that *mf*-Lon is not able to degrade RscA, an *E. coli* Lon substrate [82]. It is still unclear if, and if so, how much the *mf*-Lon can degrade *ec*-SsrA tagged proteins.

As hinted above, increasing the pool of degron sequences (especially those recognized by proteases other than ClpXP) is necessary to provide researchers with a diverse library of orthogonal degradation tags to *mix & match* on demand. In that regard, the set of N-terminal sequences recognized by ClpAPS in native *E. coli* proteins holds a clear potential to develop synthetic circuits that remains largely unexplored [126,127]. Newly produced tags based on N-degrons have been used in a few circuits [15,50,56,105]. In these systems, the degradation strategy consists of fusing the protein sequence to a N-degron sequence separated by a viral protease cleavage site. As a result, a stable protein is produced in the absence of the viral protease because the N-degron is protected from ClpS recognition. However, if the viral protease is expressed, it cleaves at the viral tag site. This exposes the N-degron to ClpAPS, resulting in the protein being degraded. A repertoire of Boolean-like gates were constructed using this strategy with three orthogonal viral proteases from Potyvirus (TEVp, TVMVp and SuMMVp) and the Y (YLFVQ) and F (FLFVQ) N-degron sequences [56].

The first intrinsic sequences used in complex synthetic circuits were MarA, MarAn20 and RepA70 (also called RepAn70) [43,49]. MarA is a transcription factor that regulates multiple genes involved in antibiotic-resistance pathways [128], and the N-terminus of MarA is a target of Lon [129]. The MarAn20 tag is the last 20 amino acid tag from the N-terminal of MarA. On the other hand, RepA70 is a 70 amino acid sequence from the N-terminal region of the protein RepA. The tag has a high affinity for ClpAP but a low affinity for ClpXP [130,131]. Some examples of other degradation sequences compatible with Lon and/or ClpAP include UmuD (15–29 amino acids) [132], SulA (150–169 amino acids) [133], HipB [43,134] (20 amino acids), SoxS (1–21 amino acids) [43,135] and MazE [136,137]. Some of these sequences were explored as potential alternative degradation tags for synthetic biology applications by fusing them to fluorescent proteins and monitoring protein levels over time as well as proteolytic crosstalk levels [43]. The results revealed that MarAn20 and RepA70 show higher degradation rates than the other tags tested (HipBc20, MazE, SoxSn20, RepA15 and HipB), and little crosstalk was observed between Lon and ClpAP. This makes these tags good candidates for synthetic biology applications. Degradation tags targeted to ClpXP, ClpAP and Lon are almost exclusively used in synthetic biology, but other proteases exist. We do not cover them here, but they are well explained in another review [14]. In addition, new research shows that the charge of the last amino acid on the C-terminus end of a peptide affects translation termination and likely protein degradation [138], although the main players behind are not well understood.

**Figure 3.**
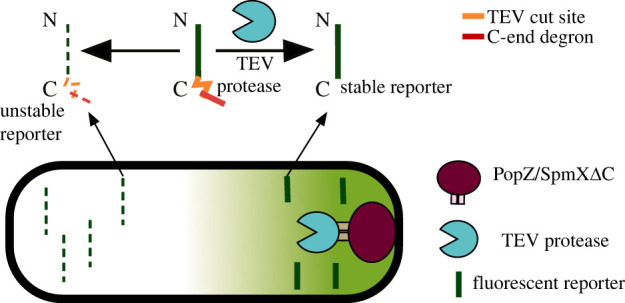
Proteases can be used to produce localized expression patterns [146]. The TEV cut site is orange and the C-end degron sequence is red. A split TEV protease (blue) is bound to the cell membrane using a PopZ-based polarity system from *C. crescentus* [147] (magenta). In the presence of the TEV protease, the C-degron is cleaved and the reporter is stabilized. In the absence of the protease, endogenous proteases recognize the C-degron and degrade the reporters. This approach creates patterns of expression in single cells. Figure adapted from [146].

Another clever use of degradation tags is employing them to create localized protein expression patterns. There is an increasing interest in developing spatial patterning strategies in bacteria [139–141], as this could give us a better understanding of fundamental biological processes from developmental biology to tissue engineering [142,143]. For example, FtsH [144] and Prc (Tsp) [145] are proteases responsible for the degradation of membrane proteins and periplasmic proteins, respectively, and hold potential for the design of membrane-localized or periplasmic circuits. Recently, Hong *et al.* [146] produced intracellular spatial asymmetries in protein production using a hybrid tag in *E. coli*. The circuit uses a split TEV protease bound to the membrane by a PopZ-based polarity system from *C. crescentus* [147] (figure 3). In the absence of the protease, the protein is degraded due to a C-degron sequence. However, in the presence of the protease located only in the cell’s pole, the C-degron is cleaved. This results in the protein levels stabilizing close to the cell’s pole and creating patterns of expression [146].

## 3. Synthetic oscillators and degradation bottlenecks

**Figure 4.**
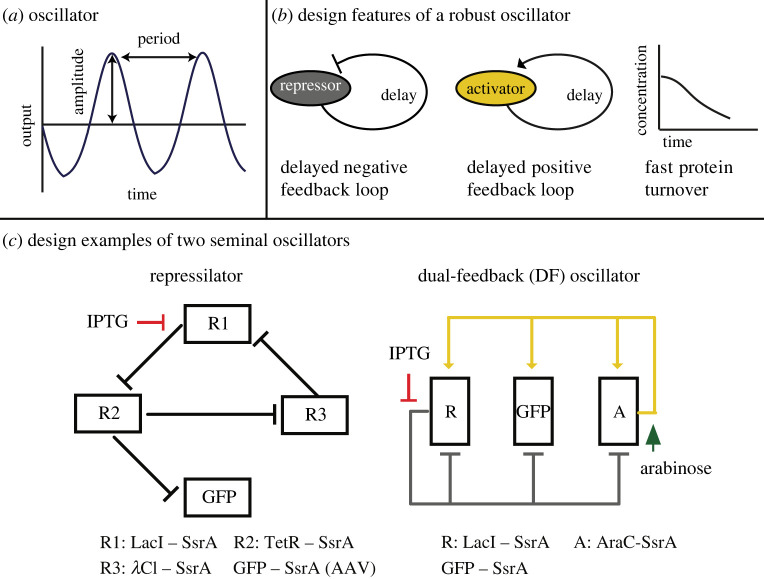
(*a*) An oscillatory output can be quantified by its period and amplitude. (*b*) The design features of a robust oscillator include a delayed negative feedback loop where a repressor (grey) represses all regulatory elements of the circuit. Mathematically, the delayed negative feedback loop is the only essential element in obtaining oscillations. It can also include a positive feedback loop where an activator (yellow) activates all regulatory elements of the circuit. It may also contain a method for rapid protein turnover such as an amino acid degradation tag, which targets proteins to a protease. Proteolytic queues can then form, which can enhance the robustness of the oscillator because the queue can add a consistent time delay to the system. (*c*) The basic design of two oscillators. Left: the repressilator [5] contains three repressors that repress each other: R1 (LacI), R2 (TetR) and R3 (*λ*Cl). Node R1 (LacI) is externally controlled with an IPTG inducer. All repressor proteins are tagged with a SsrA degradation tag (LAA), while the fluorescence reporter (GFP) is tagged with the SsrA variant (AAV). Both LAA and AAV tags are used to target proteins to be degraded by the ClpXP protease. Right: the dual-feedback (DF) oscillator [6] contains a repressor (LacI) that represses all regulatory elements, including itself (negative feedback loop), and an activator (AraC) activates all regulatory elements, including itself (positive feedback loop). Both nodes are controlled with the inducers (IPTG and arabinose). All elements are tagged with the same SsrA-tag (LAA) sequence from *E. coli*, thus the original repressilator and DF oscillator rely on the formation of proteolytic queues for oscillations.

Many natural biological systems oscillate (figure 4*a*), with examples of oscillations in all domains of life such as the cyanobacterial circadian clock [148], the bacterial cell cycle (Min oscillations) [149], the response of the tumour suppressor p53 protein in eukaryotes [150], cellular differentiation in biofilms [151] and many more. Oscillations can occur at the transcriptional and translation level such as a periodic expression of genes during the cell cycle; at each stage of the cell cycle-specific genes need to be expressed for proper cell division. These oscillations are often periodic with a regular cycle. Some natural oscillation cycles can be controlled by an aperiodic signal including non-biological [152] and biological systems [153–155]. Synthetic bacterial oscillators have been instrumental in exploring new frontiers of aperiodic controlled oscillators in biological systems [156].

Natural oscillators display three important characteristics: robustness, coherence and tunability [42]. A long-term goal of synthetic biology is to build oscillators that exhibit these properties for industrial and medical applications [7,157–165]. However, this endeavour has proven to be challenging. Bottom-up approaches have identified three key components in the core design of oscillators: (i) a negative feedback loop (essential); (ii) a positive feedback loop (enhancement of the oscillatory behaviour); and (iii) rapid protein turnover, our main focus in the context of this review. Protein turnover is not an essential component, but its incorporation has led to more robust oscillators [42,166,167] (figure 4*b*). Thus, the control of the oscillatory dynamics is often closely linked to understanding protein degradation. Several theoretical work support the importance of degradation speed in controlling oscillations [168–170].

The most commonly employed method to achieve rapid protein turnover is actively degrading transcription factors (TFs) and/or fluorescent reporters using *E. coli* SsrA tagged proteins [5,6,44]. However, the lack of orthogonal degradation mechanisms and the use of strongly induced promoters cause the formation of degradation bottlenecks (proteolytic queues) [40,51,171]. This is due to the mismatch between the cell’s relatively low number of proteases and the high number of proteins (table 1). Proteolytic queueing has been observed in wild-type bacteria during stress conditions and linked to up-regulation of the sigma factors *σ*_*S*_ and *σ*_32_ [24,174–177]. It is also associated with antibiotic survival strategies [177,178]. *In vivo* experiments showed that ClpXP often works near or in a saturated regime, and that queueing can lead to coupling between unrelated proteins targeted by the same proteolytic complex [40]. Thus, proteolytic queueing affects the dynamics and properties of synthetic oscillators. As a result, oscillators have often been designed specifically to leverage queueing, as we will discuss in this section.

**Table 1.** The average number of known proteolytic units in *E. coli* during exponential (left: growth in minimal media with glucose, right: growth in LB) and stationary phase (left: after one day in stationary phase, right: after 3 days in stationary phase). All data was obtained from dataset 2 in [172], except ClpS numbers that are from [98]. Subindexes indicate the number of units that build a functional enzyme (e.g. ClpX_6_ indicates that a functional ClpX enzyme is formed by 6 units). The numbers in the molecules per cell column reflect the total numbers of functional enzymes in a cell. Note that in bacteria, highly expressed proteins (e.g. ribosomes) are in the order of approximately 27 000 molecules/cell [173].

enzyme	molecules per cell	
	exponential	stationary
Lon	1139|3411	741|826
ClpX_6_	546|916	166|205
SspB_2_	176|356	87|52
ClpA_6_	88|246	25|26
ClpP_14_	630|865	288|312
ClpS	250|300	250|300
FtsH	2236|3956	972|924
Prc	506|621	213|166

Proteolytic queues can enhance or suppress oscillations depending on the queue size [40,171,179]. Understanding this phenomenon requires us to provide details about the design principles underlying synthetic oscillators (figure 4*b*). Generally speaking (see notable exceptions at the end of this section), bacterial synthetic oscillators share some structural features; the presence of delayed negative feedback loops and targeted degradation for rapid protein turnover [42]. Time delays occur naturally through the transcription, translation and protein maturation processes, but they can also be enhanced by proteolytic queueing. As queues build up, the amount and availability of degron-tagged repressor molecules increase, thus enhancing the delay effects [40,180,181]. Despite that delayed negative feedback loops are mathematically sufficient (and necessary) to achieve oscillations, as of yet, no robust biological oscillator has been produced that solely relies on this mechanism [42]. Most use queueing and/or positive feedback loops. Positive feedback loops are a key design feature to increase the robustness of oscillations [6]. Positive feedback loops also affect the queueing dynamics, as they lead to greater production of regulatory elements (repressors and/or activators). Regulatory elements are typically targeted to the same proteolytic complex (e.g. by the use of the same SsrA tag), resulting in the degradation queues building up faster and delays in both positive and negative feedback loops.

Our understanding of the significance of proteolytic queueing in synthetic oscillators stems from the theoretical and experimental work done with the dual-feedback (DF) synthetic oscillator [6,11]. Though the repressilator, the first experimental oscillator built [5], was produced 8 years prior to the DF oscillator and also relied on proteolytic queueing (figure 4*c*). The repressilator operates on the most basic design principle, where only negative feedback loops are involved through three mutually repressing genes (lacI, tetR, cI) tagged with an *ec*-SsrA (LAA) tag (figure 4*c*). A fluorescence gene (GFP), placed on a different plasmid, reports the dynamics of the repressor cI. However, GFP is tagged with the SsrA variant AAV, which has a slower degradation rate than the original *ec*-SsrA tag [37]. It is worth noting that in the repressilator only a small percentage of cells exhibit oscillations and is not synchronized across the population [5]. However, more cells exhibit oscillation with the DF oscillator [6] and these cells can be synchronized across the population. In this manner, the DF oscillator is more robust than the repressilator.

Notably, the DF oscillator was the first design to implement a positive-feedback loop along with a negative-feedback loop (figure 4*c*). In the DF-design, all genes of the circuit include the same version of the *ec*-SsrA tag, and it uses the hybrid promoter *P*_lac/ara_ [182]. This promoter is negatively controlled by LacI and positively controlled by AraC. The oscillator can be externally regulated using IPTG and arabinose. IPTG leads to an increase in LacI, the repressor, while arabinose leads to an increase in AraC, the activator. Hence, the oscillator can be externally regulated in a dose-dependent manner using IPTG and arabinose. The DF oscillatory dynamics is preserved at different temperatures, several growth media and usable in multiple organisms with only minor changes [6,109]. Furthermore, a modified version of the DF design (the quorum oscillator) that exploits cellular entrainment (discussed below) via a quorum sensing mechanism reduces cell-to-cell variability in the output [44]. In the DF and quorum oscillator, all genes of the circuit include the same version of the *ec*-SsrA tag (figure 4*c*); thus, the dynamics of this oscillator is affected by proteolytic bottlenecks as well.

**Figure 5.**
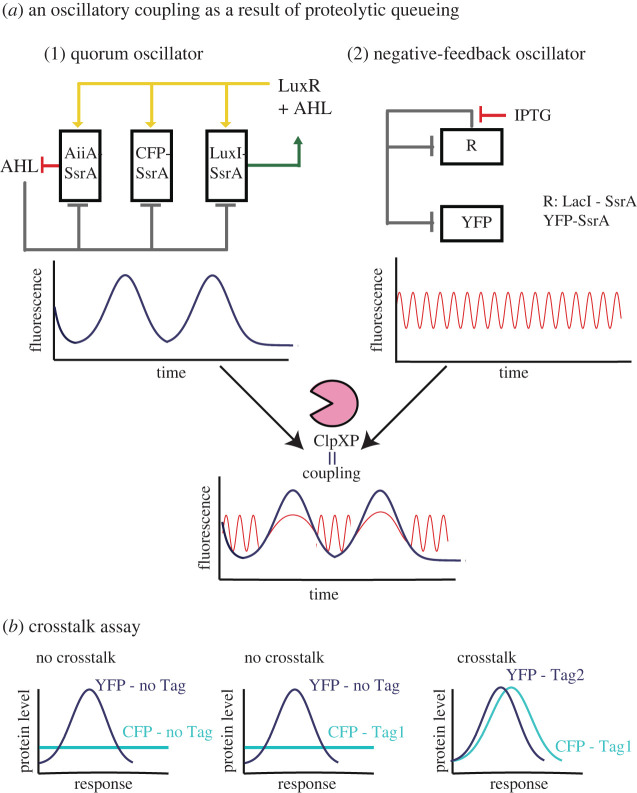
(*a*) Sharing enzymatic resources can lead to coupling events between two independent oscillators. The output of two otherwise independent oscillators can be synchronized via the same degradation tag (*ec*-SsrA) [11]. Both oscillators show independent oscillations on their own, but when co-expressed in the same host, their period and amplitude synchronize because of the shared enzymatic machinery for degradation (ClpXP). Coupling arises through a proteolytic bottleneck. (*b*) The Crosstalk Assay [43] allows quantification of the level of crosstalk between two independent degradation tags. The behaviour of two independent fluorescence reporters (CFP and YFP) can be investigated using degradation tags. CFP derivatives are expressed at a constant level. If there is no crosstalk, the induction of YFP can lead to no change in CFP (left and middle). However, if there is crosstalk induction of YFP containing a degradation tag will lead to an increase in CFP (right). This indicates that crosstalk occurs at the protease level because CFP expression is constant. CFP and YFP do not act as transcription factors, and fluorescence crosstalk is only detected when proteins contain degradation tags (right).

A particular advantage of using oscillators that use proteolytic queueing is that a population of cells can be entrained (i.e. synchronized by an external signal) [51,170]. Entrainment allows coherence, cells follow the frequency (similar amplitude and period) of the external signal synchronizing their behaviour. The classical example of entrainment is in humans where the circadian rhythm (the process that regulates the sleep–wake cycle to the planet’s 24 h rotation) is entrained by the position of the sun in the sky [183]. Proteolytic queueing and the process of entrainment has been exploited as a design feature in synthetic oscillators. The DF oscillator was entrained via proteolytic queueing by using a fluorescent protein-tagged targeted to ClpXP [51]. The fluorescent protein (CFP-SsrA) and the DF oscillatory proteins are all targeted for degradation by ClpXP. The CFP-SsrA was induced by an external AHL signal, and the DF oscillator was controlled via IPTG and arabinose (GFP-SsrA as fluorescent reporter). The transcription of these two circuits were independent of each other, but connected through the same proteolytic pathway (ClpXP). When the level of CFP-SsrA was modulated in a microfluidic device using AHL, the GFP-SsrA output followed the CFP-SsrA oscillation periods. This resulted in the entrainment of the oscillator where thousands of cells followed the external AHL signal (CFP-SsrA). This study demonstrated the power of coupling unrelated networks (only related through a proteolytic queue) to get controllable single-cell and population dynamic responses [51]. In another study, entrainment via proteolytic queueing was exploited to couple the output of two independent oscillators [11]: the DF oscillator [6] and the quorum oscillator [44]. By modifying the DF oscillator so it lacked the positive feedback loop (LacI-SsrA and YFP-SsrA), its output was synchronized with the quorum oscillator (AaiA-SsrA, LuxI-SsrA, CFP-SsrA) [11] (figure 5*a*). Under these scenario, each circuit produced independent oscillatory dynamics on their own. When combined in the same host, a strong coupling and synchronization of the oscillations occurred orchestrated through proteolytic queueing [11]. All proteins contained the SsrA-tag, which targeted the proteins to the same degradation machinery, ClpXP. As a result, the coupled oscillator displayed a greater coherence than the individual oscillators [11]. Also, as proteolytic queues are susceptible to changes in protein levels, the couple oscillators can detect small changes in the input signal, showing an increased sensitivity. In a further study, a method was developed to quantify the level of proteolytic crosstalk between independent proteins tagged with degrons. This assay, the Crosstalk Assay [43], is helpful for quantifying coupling due to proteolytic queues and to identify degradation tags that are not coupled, and show potential for the construction of orthogonal circuits (figure 5*b*).

The last couple of examples leveraged coupling through queues to synchronize oscillators; however, coupling can be a major limitation when designing other circuits. Proteolytic bottlenecks are a major impediment to modulate independently the amplitude and period of the oscillations [179]. This can be a substantial shortcoming since building tunable oscillators is a sought-after property in synthetic designs [184]. To modulate amplitude and period independently, Tomazou *et al*. [179] have proposed alternative designs that reduce queueing (by tweaking the expression levels of proteases or by using orthogonal degradation tags). While most oscillators build to date exploit degradation tags (and hence, are prone to proteolytic queueing), it is important to highlight some recent circuit design solutions that minimize or avoid degradation tags and proteolytic queues. A variation of the repressilator without SsrA tagging [185] used ‘sponge elements’ (additional TF binding sites) [186] to reduce the availability of free transcription factors. The sponge elements represent an alternative way of increasing the turnover rates of regulatory elements. They can be used to decouple the oscillatory output from cell division in the absence of degradation tags. This modified repressilator [185] showed oscillations both in the absence and presence of sponge elements but with an increased period compared to the original repressilator. The disadvantage of using sponge elements over degradation tags is that shorter periods has not been achieved (more practical oscillation periods are required for applications such as biosensors). *Escherichia coli* can double every 20 min, and feedback can be quick with oscillators that use degradation tags such as the DF oscillator (which can oscillate in less than 20 minutes). However, the modified repressilator takes several generations to give an oscillatory output; 10 and 14 generations to oscillate with and without sponge elements, respectively. The use of degradation tags allows the DF oscillator to function independently from the cells doubling time; however, the modified repressilator is still dependent on the cells doubling time for removal of proteins.

A new family of synthetic circuits based on CRISPR interference has emerged that are notably relatively independent of proteases to modulate the turnover rates of the regulatory elements [49,187–189]. These circuits rely on CRISPR nucleases variants (mainly dCas9) that bind to the DNA without cleaving it (nuclease-null). dCas9 binding is guided by an associated RNA molecule (gRNA). These effector molecules replace the role of natural TFs in synthetic circuits [188]. A modified repressilator was designed that uses the dCas9 protein to replace the role of LacI in the original design [187]. A single-guide RNA (sgRNA) binds to the *P*_lac_ promoter and imitates the repressive function of LacI (negative feedback loop). In the dCas9-repressilator design, the turnover of TetR and *λ*CI are still dependent on the ClpXP proteolytic complex as they harbour a SsrA-tag. However, dCas9 is untagged and the sgRNA turnover rate depends on RNAses instead of proteases (as well as on dilution due to cell growth) [187]. Oscillations in the dCas9-repressilator have a longer period (avg. period 11.7 ± 0.4 h) [187] than the original repressilator (avg. period 2.7 ± 0.7 h) [5]. Adding sponge elements provided extra binding sites for sgRNA and dCas9 to reduce the period; however, the period was still much greater than the doubling time of *E. coli* [187]. This highlights the importance of tightly controlling the number of regulatory elements in the cell.

The CRISPRlator is another new design that follows the original repressilator framework but is entirely based on the CRISPRi system [49]. The CRISPRlator uses three sgRNAs (with different binding affinities) to control their expression and three fluorescent reporters: mCherry, mCitrine and Cerulean. They are tagged with MarA, MarAn20 and RepA70 degradation tags, respectively [43]. This circuit is the only current synthetic oscillator that does not rely on the SsrA tag, combines different degradation tags, and thus does not rely on crosstalk via proteases between each tagged element. No crosstalk should occur between tagged proteins because different proteolytic pathways process them. As a result, in the CRISPRlator, the cell growth and the turnover rates of sgRNAs are the time scales that drive the period of the oscillations. As for the robustness of these designs, the CRISPRlator, to cite an example, shows long-term and synchronous oscillations, which the authors hypothesize stems from the robust inheritance of the oscillatory state across cell divisions [49].

## 4. Modelling frameworks to understand the degradation dynamics

### 4.1. A brief introduction to the mathematics of queueing theory

Synthetic biology does not just rely on hands-on engineering to implement cell functionalities. Providing a mathematical/modelling framework is equally important to, for example, check if the design expectations are fulfilled. Queueing theory (or the theory of waiting lines) was developed to study, probabilistically, the problem of customers (in a general sense) waiting in line to be served. Hence, it is a framework particularly fit to understand/model proteolytic queueing. Queueing theory was introduced by A. K. Erlang in 1909 to address waiting time problems in telephone networks due to a limited number of servers [190]. Its development has been progressive since then motivated, in part, by the need for solving practical problems across different disciplines (traffic, business, manufacturing and computer systems) [191–194]. Surprisingly, with very few exceptions [41,52,195], the usage of queueing theory to study degradation bottlenecks is anecdotal despite its potential.

The basic elements of a system affected by queueing consist of a ‘customer’ and a ‘server’. In the context of a degradation process, the customers are the proteins waiting for degradation and the servers are the proteases [40]. In general, a queueing process is characterized by the following steps/features [196]. (i) Customers’ arrival, which describes how customers are added to the queue. Customers that arrive randomly can be described by the characteristic random time interval between two consecutive arrivals (conventionally, *λ* is used to represent the arrival rate). (ii) Serving process, which represents how the customer that waits in the queue will be served. In queueing modelling, the actual physical process happening at the server is usually ignored, as the key information is the amount of time that a server needs to serve the customer. Typically, *μ* is used to describe the serving rate (number of customers that are served during a time interval). (iii) The capacity of a system that indicates the maximum number of customers that a system can hold. Note that the system capacity includes not only the number of customers waiting in the queue but also the customers being served. (iv) The queue discipline, which accounts for the specific rules of a server to accept customers. Queueing disciplines include scenarios such as first-come-first-serve (FCFS), last-come-first-serve (LCFS), random selection for service (RSS), etc. [197–199].

**Figure 6.**
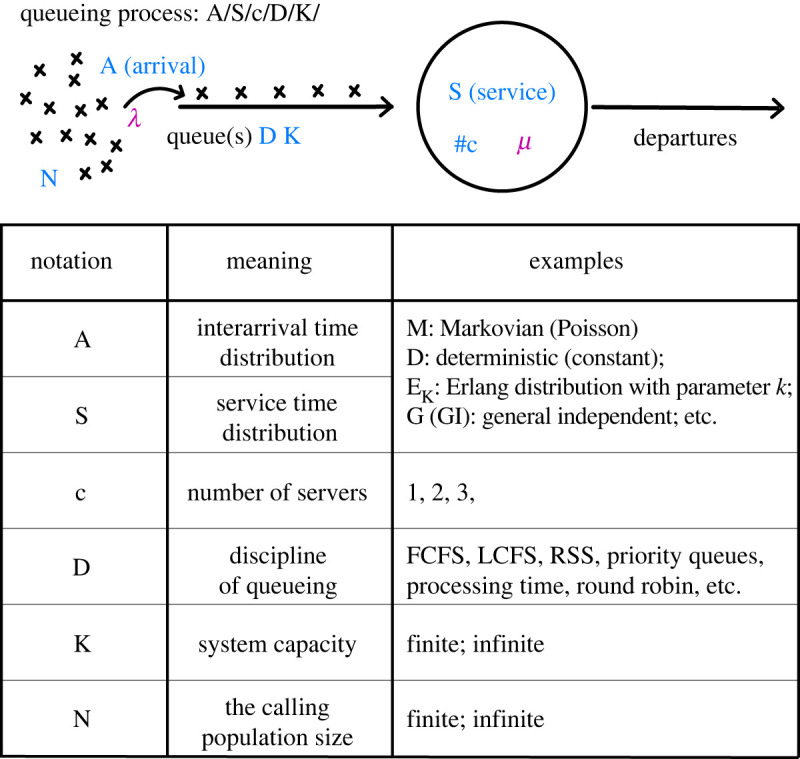
The process of queueing and its corresponding notation. Notations in a queueing process *A*/*S*/*c*/*D*/*K*/*N* are marked in blue in the schematic diagram (top) and are explained in the table (bottom). *λ* and *μ* (purple) represent the customer arrival rate and server processing rate, respectively.

In order to describe the queueing features, Kendall [200] introduced in 1953 a notation that still applies: *A*/*S*/*c*. Where *A* and *S* represent the inter arrival and service time distributions, respectively, and *c* stands for the number of servers. Eventually, Kendall’s notation was further extended to include additional information: *A*/*S*/*c*/*D*/*K*/*N*, in which *D*, *K*, *N* represent the queueing discipline, the system capacity and the arriving population size, respectively (figure 6). For example, using the basic Kendall’s notation a simple queueing model would read *M*/*M*/1. Where *M* (Markovian) indicates that the arrival and service time distributions of a single (1) server follow a memory-less random process. Such a system is in fact equivalent to a regular birth–death process [201].

The ratio between the customer arrival rate and the server process rate is typically denoted by *ρ* = *λ*/*μ*, which indicates the fraction of time the server is being used by the arriving customer. If the system has an infinite queue and *ρ* < 1, it can be shown that the steady-state probability of having *n* customers in the queue reads [202]

4.1 $$P_{n}=(1-\rho )\rho^{n}=P_{0}\rho^{n},$$

where *P*_0_ = 1 − *ρ* represents the probability of having no queueing customers. Consequently, the average number of customers in the system is

4.2 $$L_{s}=\sum_{i=0}^{\infty }iP_{i}=\frac{\rho }{1-\rho }.$$

Little’s equation, *L*_*s*_ = *λW*_*s*_, is particularly a handy formula since it relates the average number of customers, *L*_*s*_, with the average time spent per customer in a (stable) queueing system, *W*_*s*_ [203]; where the average time per customer spent reads,

4.3 $$W_{s}=\frac{1}{\mu -\lambda }.$$

**Figure 7.**
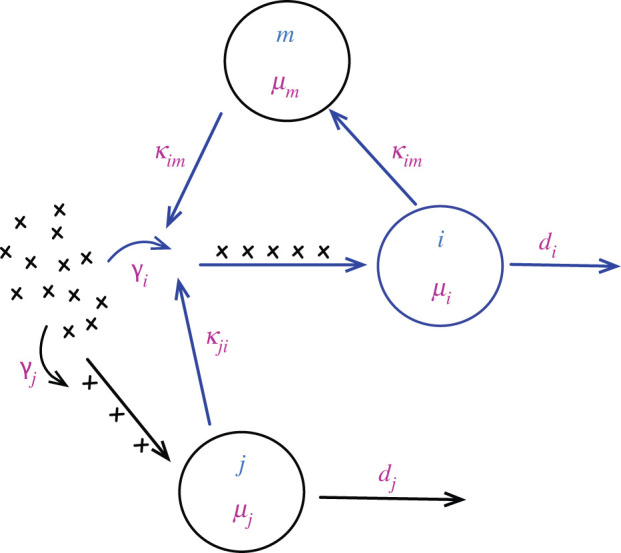
An illustrative model of the Jackson’s network. The servers are denoted by circles. The queue at each server may form due to customers coming from the outside population and customers who finish the service at other servers. That is, once the customers are done with the service at a server, they either choose to leave the system or join the queue of another server.

When there is more than one type of server, customers leaving one server may join the queue of another server. In the context of proteolytic bottlenecks, this is a situation that is difficult to imagine (since once a protein is ‘handled’ by a protease it will not be further processed). Still, depending on the cellular resources, queues can also develop in the course of transcription, translation and during post-/trans-translation modification (see next section). Consequently, it is worth to revisit some useful concepts of multi-server queueing. In this case, queueing networks are formed with topologies that can differ depending on the systems that is being studied. Owing to their increasing complexity, analytical results of queueing networks are difficult to obtain. A exception to this rule is the so-called Jackson’s network [52]. Jackson’s network consists of *N* single-class FCFS queueing systems where the distributions of the inter-arrival and service times are assumed to be exponential with constant average rates (figure 7).

If *γ*_*i*_ denotes the (external) arrival rate to the server *i* and *κ*_*im*_ is the transition probability for a customer finishing the service at *i* and joining the queue to the server *m*, the total arrival rate to the server *i* is,

4.4 $$\lambda_{i}=\gamma_{i}+\sum_{m\neq i}^{N}\lambda_{m}\kappa_{mi}.$$

Also, the probability to leave the system through the server *i* is $d_{i}=1-\sum_{m\neq i}^{N}\kappa_{im}$. Further, the stationary probability of each server to have *n*_1_, *n*_2_, …, *n*_*N*_ customers is,

4.5 $$P(n_{1},n_{2},\ldots ,n_{N})=\prod_{i=1}^{N}(1-\rho_{i})\rho_{i}^{n_{i}}=\prod_{i=1}^{N}P_{n_{i}},$$

where *ρ*_*i*_ = *λ*_*i*_/*μ*_*i*_ and $P_{n_{i}}$ stands for the stationary probability of the server *i* to have a queue of *n*_*i*_ customers.

Depending on the number of ‘customer’ types, queueing models are categorized into single- or multi-class. In the former, there is only one type of ‘customer’, whereas in the latter different types of ‘customers’ are present in the queues. The aforementioned *M*/*M*/1 queueing model and Jackson’s network are single-class. Single-class models have been used to describe the lac operon and metabolic pathways [52,204]. However, in the context of proteolytic bottlenecks, most models are considered multi-class as shown below [41,52,205].

### 4.2. Theoretical models of proteolytic queueing

As mentioned above, many cellular processes, including transcription, translation and degradation, involve a competition for limited resources. For instance, DNA binding sites compete for a limited number of transcription factors (TFs); RNAs queue up for a limited pool of ribosomes or miRNAs; and proteins compete for proteases (e.g. SsrA tagged proteins competing for ClpXP) [11,47,206]. All these processes can be analysed and modelled from the viewpoint of queueing theory. Obviously, modelling the degradation dynamics differs significantly from other cellular processes but, in general, queueing models can be adapted to account for any process where ‘customers’ wait for ‘services’ provided by a limited amount of ‘servers’. And yet, the question of how ‘customers’ interfere/interact with each other, and how cells exploit queueing to achieve either efficiency or specific cellular functions, remains largely unexplored.

Within the specific context of enzymatic degradation, Mather *et al.* proposed a seminal study that addressed competition of different proteins for a common protease (*E. coli*’s ClpXP) by using a multi-class queueing model [181].

Thus, this study considered the scenario of cells with *m* protein species, *X*_1_, …, *X*_*m*_, produced at rates *λ*_1_, …, *λ*_*m*_, from their corresponding mRNA templates, *D*_1_, …, *D*_*m*_, and that are enzymatically degraded by a common pool of a protease species, *E*, that is limited in terms of its numbers, *L*. In terms of biochemical reactions such a process reads

$$\begin{array}{rl} & D_{i}\overset{\lambda_{i}}{⟶}X_{i}+D_{i}\\ & X_{i}+E\overset{\eta }{⟶}X_{i}E\\ & X_{i}E\overset{\mu }{⟶}E. \end{array}$$

In the model, the binding rate of the proteins to the enzyme, *η*, is assumed to be constant—as well as the protein degradation rate, *μ*, that is supposed to be protein independent—and the chemical reactions follow a Poisson process. Further, dilution effects due to cell growth and division are also considered (at a rate *γ*),

$$X_{i}E\overset{\gamma }{⟶}E$$

and

$$X_{i}\overset{\gamma }{⟶}O.$$

**Figure 8.**
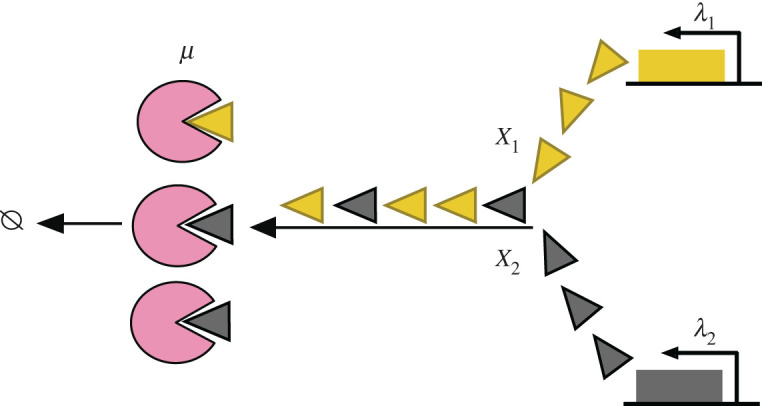
A schematic representation of a multi-class proteolytic queueing model, adapted from [181]. Proteins *X*_1_ (yellow) and *X*_2_ (grey) are produced from two independent transcriptional processes, but are being degraded by a common enzyme type (pink). When the enzymes (servers) are fully occupied, a queue with multiple classes of proteins (customers) is formed. Note that proteins join the queue randomly. The queue length depends on the interplay between the enzymatic processing rate and the protein arrival rate to the queue.

That is, proteins are removed from the system either by the enzymatic degradation machinery or by the growth-division dilution process. By considering two protein species (figure 8), the study found that at the steady-state, and assuming a small dilution rate (*γ* ≪ *μ*), the correlation between the number (or the concentration) of the two protein species peaks around a balanced point, in which the total production rate is balanced with the enzymatic degradation rate: *λ*_1_ + *λ*_2_ = *Lμ*. When the enzyme works either in an underloaded regime, i.e. *λ*_1_ + *λ*_2_ < *Lμ* (such that protein production is slower than the degradation: no queue), or when the enzyme is overloaded (faster production than degradation: queue formation), the correlation between the number of two protein species in the queue decreases with respect to that at the balanced point.

While this study introduced first the competition of different proteins for a common protease, it did not consider how the presence of multi-proteases (i.e. multi-servers) affects the dynamics. To address this point, a multi-protease queueing model was later proposed [41]. This model follows the same scheme but, in this case, the degradation by different classes of enzymes was introduced:

$$X_{\;j}+E_{i}\overset{\mu_{ij}}{⟶}E_{i},$$

where *μ*_*ij*_ represents the degradation rate of substrate *j* being processed by the enzyme class *i*. This theoretical study was also one of the first that coupled the multi-proteases degradation queueing system with a protein production oscillatory network. The study suggests that the bottleneck on multi-protease networks still allows a substantial coupling in the dynamics of proteins even between substrates with considerable differences in their binding affinities to the proteases. Therefore, queueing models support the experimental observations showing that the proteolytic bottlenecks lead to upstream coupling of independent pathways [51].

Finally, on the modelling side, it is worth mentioning recent efforts to consider the effect of the competition for degradation by different proteins by using a phenomenological approach [207]. In that study, following [11], the authors modelled the concentration (*ρ*) dynamics of a pair of proteins, *X*_1_ and *X*_2_, that are degraded by the same protease by means of a system of ordinary differential equations:

$$\frac{d\rho_{X_{1}}}{dt}=F_{1}(\rho_{X_{1}},\rho_{X_{2}})+\rho_{X_{1}}\Gamma (\rho_{X_{1}},\rho_{X_{2}})$$

and

$$\frac{d\rho_{X_{2}}}{dt}=F_{2}(\rho_{X_{1}},\rho_{X_{2}})+\rho_{X_{2}}\Gamma (\rho_{X_{1}},\rho_{X_{2}}),$$

where *F*_*i*_ are the functions that account for the production and regulatory interactions of protein *i*, and $\Gamma (\rho_{X_{1}},\rho_{X_{2}})=$ $a/(b+\rho_{X_{1}}+\rho_{X_{2}})$ describes effectively the degradation and includes competition effects. Thus, if the concentration of proteins is ‘low’ with respect to the processing capacity of the protease (i.e. $\rho_{X_{1}},\rho_{X_{2}}\ll b$) the degradation terms tends to the regular exponential decay. However, if the protease is saturated (i.e. $b\ll \rho_{X_{1}},\rho_{X_{2}}$) the degradation leads to coupling effects. The study showed that such a coupling between proteins can be leveraged to engineer self-organized criticality in cells.

## 5. Conclusion

Even though advances have been made in the discovery of new degradation tags for bacterial synthetic circuits, there is still a considerably lack of available tags. Leveraging a library of novel protein degradation tags is necessary to be able to build complex and orthogonal synthetic gene networks. Just as native systems exploit orthogonal degradation pathways to avoid coupling between genetic networks, synthetic biology should aim to exploit a similar strategy when building multiple circuits to be used together. To date, most synthetic circuits use almost exclusively *E. coli’s* SsrA tag [54] for targeted degradation, but alternative degrons are starting to be explored. In particular, N-degrons [20], or intrinsic degrons [25,43] are providing excellent pools of sequences with great potential to be used in synthetic biology [43,49,50]. The use of viral proteases along with native bacterial proteases [56] or degrons/proteases from evolutionary distant bacterial species [48] are other promising alternatives for tightly controlling protein levels. Coupling is not only a consequence of the redundancy in degradation pathways, but also due to bottlenecks in degradation processes (proteolytic queues) that result from the low numbers of proteases available in the cell [40]. Queueing has been successfully exploited to intentionally couple the output of synthetic oscillators, but also to entrain (i.e. synchronize by an external signal) the oscillatory output of thousands of single cells [41,51]. This has potential, for example, in the development of robust biosensors with applications in industry and medicine [208–210]. Proteolytic queueing is also known to enhance time delays of transcription factors which is a key feature for synthetic oscillators to display robustness (a sought after property in these types of circuit topologies) [170]. However, queues can also suppose a nuisance in terms of reliability and independent control of amplitude and period in oscillators [179]. As a result, there has been an increasing trend in the field where researchers have started to engineer away degradation tags [185,187]. This approach comes with a price, as the circuits built are slower and less tunable compared to those that rely on proteases. They do also depend on the host’s growth rate for protein removal via dilution, thus inhibiting oscillating periods less than the doubling time of the cells.

There is a need in the field to better characterize the exact molecular players (proteases and chaperones) involved in the recognition of degrons. One way to do so could be to deconstruct degradation tags by mutating specific residues and use experimental (e.g. the crosstalk assay [43]) and theoretical approximations to understand degradation and queueing dynamics in the different mutants. Such approaches could have the potential to provide the synthetic biology community with new libraries of degradation tags with different degradation rates and sensitivities to queue formation to *mix & match* on demand.

On the theoretical side, developing better quantitative models that take into account proteolytic queue effects to understand circuit dynamics is also important. Here we have introduced notions of formal theory traditionally applied to computer networks or call centres that we hope will help researchers introduce these concepts. Some theoretical work based on queueing theory has already shown how crosstalk can couple free-running independent oscillators [41], but there are still other aspects such as stochastic effects in queue formation or entrainment that could be implemented in the theoretical frameworks. Combining queueing theory and experimental work in the study of degradation pathways will accelerate our understanding of the effects of degradation dynamics in synthetic circuits and guide the design of new experiments.

Herein we have covered the use of degrons for programmable protein degradation in bacteria. We would like to highlight that the use of eukaryotic degrons and viral proteases have also been implemented to design synthetic circuits in eukaryotic systems [15,17,211–213]. Moreover, in mammalian cells targeted degradation makes possible to eliminate specific proteins at will, thus opening extensive therapeutic opportunities. For example, some drugs (currently in clinical trials) use this technology to treat cancer patients [214].

All in all, over recent years, it has become evident that degradation control is a fundamental aspect in the design of synthetic circuits that opens new possibilities for programmable control of circuits at the protein level and is also paving the way to develop new biomedical therapies.

## Data Availability

This article has no additional data.
